# Comprehensive prediction of multiple low temperature and high humidity diseases in greenhouse cucumbers based on infection suitability index

**DOI:** 10.3389/fpls.2026.1809965

**Published:** 2026-08-19

**Authors:** Zhulin Li, Yumeng Lu, Chang Liu, Zhongqiu Li, Shuhan Zhou, Juanjuan Ding, Tianlai Li

**Affiliations:** 1 College of Horticulture, Shenyang Agricultural University, Shenyang, China; 2 Key Laboratory of Protected Horticulture, Ministry of Education, Shenyang, China; 3 National & Local Joint Engineering Research Center of Northern Horticultural Facilities Design & Application Technology (Liaoning), Shenyang, China

**Keywords:** cucumber diseases, environmental monitoring, infection suitability index, prediction model, warning system

## Abstract

Facility agricultural greenhouses are prone to forming low-temperature and high-humidity environments, which frequently induce the concurrent outbreak and spread of fungal diseases such as downy mildew, gray mold, and black spot disease. However, existing predictive studies often focus on individual diseases or environmental factors, making it difficult to meet the precise disease control needs in production settings with complex diseases. To address this issue, this study constructs an Infection Suitability Index (ISI) that quantifies humidity suitability using Beta functions and piecewise linear functions, enabling nonlinear coupling of temperature and humidity. With this as the core input, multiple linear regression (MLR), support vector regression (SVR), and MLR-SVR hybrid predictive models are developed. The results indicate that ISI is significantly negatively correlated with the incubation period of all three diseases. The MLR-SVR hybrid model demonstrated the best prediction performance, with a coefficient of determination (R^2^) ranging from 0.962 to 0.982 and prediction accuracy between 88.5% and 92.3%. It shows excellent applicability in greenhouse environments. This study provides a new method for multi-disease collaborative early warning in facility cucumber cultivation, and the proposed ISI index along with the hybrid model system has important practical value for intelligent pest control and precision agriculture.

## 1 Introduction

Protected agriculture is a key medium for ensuring the year-round balanced supply of vegetables. However, the greenhouse environment, characterized by its relatively enclosed structure, limited ventilation, and susceptibility to external climatic influences, often creates microclimatic conditions with low temperatures and high humidity. These conditions provide a favorable environment for the occurrence and spread of various fungal diseases. Cucumber, one of the most widely cultivated vegetable crops in China’s protected agriculture, is commonly affected by the compound damage caused by three typical low-temperature and high-humidity diseases: downy mildew, gray mold, and black spot disease. These diseases are characterized by short incubation periods, rapid spread, high epidemic risks, and narrow windows for control, which can lead to yield losses of 20%-50%. In severe cases, entire plants may die, and yield reductions can be substantial. These diseases have become a key limiting factor for ensuring high-quality and stable cucumber production in protected agriculture and for implementing green pest control (Liu et al., 2022; Shi et al., 2022; Zhang et al., 2024).

Epidemiological studies have shown that the synergistic coupling of temperature and relative humidity is the core driving factor regulating the processes of spore germination, invasion, colonization, and incubation of pathogens. All three diseases exhibit a high dependence on temperature and humidity conditions: downy mildew has an optimal growth temperature of 16–24 °C, with infection efficiency significantly enhanced when the relative humidity exceeds 85%; gray mold is most likely to outbreak under conditions of 15–22 °C and near-saturated high humidity; black spot disease has an optimal infection temperature range of 17–25 °C and is also highly sensitive to high humidity environments (Sun et al., 2017a). A single environmental factor is insufficient to accurately reflect the actual infection potential of the diseases, while the dynamic combination of temperature and humidity directly determines the speed and severity of disease outbreaks.

In recent years, classical temperature and humidity suitability models used for plant disease early warning have been widely applied, including the Thom index, Hutton index, and temperature-humidity suitability weighted model. These models typically use linear weighting of temperature and humidity, empirical threshold determination, or simple function fitting. By allocating fixed weights, they provide risk assessments, offering important references for predicting individual diseases (Neufeld et al., 2017; Sun et al., 2017a; Zhong et al., 2020; Castaño-Serna et al., 2025). However, these methods have obvious limitations: their mathematical structure mainly relies on linear superposition, without considering the nonlinear coupling effects between temperature and humidity; many parameters depend on empirical settings, lacking a physiological-ecological foundation for the pathogens; and the models are typically designed for single diseases, making it difficult to meet the unified prediction needs for multiple concurrent diseases (Cao et al., 2023, Cao et al., 2025; Li, 2025).

To address the aforementioned scientific issues and application limitations, this study constructs an Infection Suitability Index (ISI) based on the physiological and ecological mechanisms of pathogen infection. It uses a Beta function to quantify the temperature suitability sub-model, a piecewise linear function to quantify the humidity suitability sub-model, and implements the coupling of temperature and humidity in a multiplicative form. Compared to traditional indices, ISI differs in its mathematical structure by shifting from ‘linear weighting’ to ‘nonlinear multiplicative coupling’; in its parameter sources, from ‘empirical settings’ to ‘experimental fitting based on pathogen-specific base temperature points’; and in its application scope, from ‘single disease-specific’ to ‘a general framework with replaceable parameters.’ These distinctions make it significantly different from traditional temperature-humidity suitability models in both structural design and biological significance. In terms of prediction algorithms, multiple linear regression (MLR) can clearly identify the linear effects of environmental factors but struggles to fit the nonlinear features of disease infection processes; support vector regression (SVR) excels at nonlinear fitting but has limitations in terms of mechanistic interpretability. Therefore, this study adopts an MLR-SVR hybrid model, using MLR to extract the linear trends between the environment and disease, and SVR to fit the nonlinear residuals and correct the predictions, balancing prediction accuracy, stability, and biological interpretability (Equations 1, 2). This approach is more suitable for unified predictions of multiple diseases (Guo et al., 2021). The study focuses on the compound diseases of downy mildew, gray mold, and black spot disease in facility-grown cucumbers, systematically constructing a temperature-humidity coupled Infection Suitability Index and validating its biological effectiveness. By using ISI as the core input, a unified prediction model for multiple diseases is developed, revealing the quantitative driving mechanisms of temperature-humidity coupling on disease infection. The results improve the prediction accuracy and practicality under multi-disease co-occurrence scenarios, providing a theoretical basis and technical support for intelligent early warning, precise control decision-making, and green, efficient production in facility cucumber cultivation (Liu et al., 2020).

## 2 Materials and methods

### 2.1 Experimental materials

The experiment was conducted from September 2023 to December 2024 at the No.25 solar greenhouse (N41°48′, E123°33′) of the Horticultural College’s research base at Shenyang Agricultural University and the Tianrun Garden artificial climate cultivation box laboratory (Equation 3). The cucumber variety used for the experiment was ‘Jinyan No.4’ (*Cucumis sativus L.*), and the cultivation substrate was a mixture of perlite, vermiculite, and peat moss in a volume ratio of 1:1:2. Seedling cultivation began on September 20,2023. When the seedlings reached the two-leaf-and-heart stage, they were transplanted into the greenhouse cultivation troughs. During the entire seedling phase, conventional water and fertilizer management was applied without the use of fungicides to ensure plant health and prevent disease, providing uniform test materials for subsequent inoculation and field trials.

The tested pathogens included cucumber downy mildew (*Pseudoperonospora cubensis*), gray mold (*Botrytis cinerea*), and cucumber black spot disease (*Cladosporium cucumerinum*). After isolation, purification, and identification, the gray mold and black spot disease pathogens were stored at low temperatures on PDA slants (*Potato Dextrose Agar Slant*). The downy mildew pathogen, being an obligate parasite, was collected from naturally infected cucumber plants in the greenhouse. Fresh infected leaves were collected, washed, and the spore suspension was prepared for live inoculation.

### 2.2 Experimental design and environmental control

#### 2.2.1 Artificial climate chamber simulation experiment

A controlled experiment was conducted using an RXZ-500D intelligent artificial climate chamber (Ningbo Jiangnan Instrument Manufacturing Co., Ltd., Ningbo, China). The chamber was operated under a 14 h light/10 h dark photoperiod. The default cultivation light settings provided by the manufacturer were maintained throughout the experiment and remained constant during the entire study period. The chamber had an effective volume of 500 L and allowed precise control of temperature and relative humidity.

Five temperature gradients (14, 18, 22, 26, and 30 °C) and five relative humidity gradients (60%, 70%, 80%, 90%, and 100%) were established, resulting in a total of 25 temperature–humidity treatment combinations. The temperature and relative humidity control accuracies were maintained within ±0.5 °C and ±5%, respectively. These treatment combinations covered the full environmental suitability ranges for the occurrence of the three target diseases.

Healthy functional leaves located at the third to sixth leaf positions in the middle canopy of cucumber plants at 30 d after transplanting were selected as inoculation materials. Leaves with uniform age and growth status were used throughout the experiment (Equations 4–6). A spore suspension at a concentration of 1 × 10^6^ spores mL^-^¹ was applied by spray inoculation, with 2 mL of inoculum uniformly sprayed onto the abaxial surface of each leaf. Following inoculation, the leaves were transferred to the climate chamber and maintained under the designated environmental conditions. The appearance of initial disease symptoms was monitored and recorded daily at 09:00 h.

To maintain the physiological activity of detached leaves during the multi-day observation period, humid incubation and supplementation with sterile water were employed to preserve leaf viability and prevent the influence of premature tissue senescence on disease development. For each disease, three biological replicates were established under each temperature–humidity treatment combination, with 30 leaves per replicate, resulting in approximately 5,400 leaves observed in total. Detailed temperature and humidity treatment parameters for the artificial climate chamber experiment are provided in **Supplementary Table 1**.

To minimize experimental bias, healthy cucumber leaves with uniform growth status were randomly selected from different plants prior to inoculation. Leaves were randomly assigned to the 25 temperature–humidity treatment combinations, and all treatment groups were subjected to identical experimental procedures and management practices.

For each treatment, 30 detached leaves were used per replicate, resulting in 90 leaves per temperature–humidity combination for each pathogen. Disease symptoms were recorded daily for 7–10 days post inoculation until stable symptom expression was observed. Detached leaves were maintained in sealed humid chambers with periodic sterile water supplementation to prevent dehydration and maintain physiological activity. Leaf viability was monitored visually and leaves showing severe senescence were excluded from analysis.

#### 2.2.2 Greenhouse field experiment

A15 m² (3 m × 5 m) isolated experimental plot was established in a solar greenhouse at the research base (Equation 7). A 1 m buffer row was maintained between adjacent plots to prevent cross-contamination and disease spread among treatments. Each treatment consisted of three replicate plots, which were analyzed as independent statistical units.

Cucumber seedlings were transplanted at a row spacing of 60 cm and a plant spacing of 30 cm. Conventional irrigation and fertilization practices were applied throughout the growing period, and no fungicides were used. The greenhouse was operated under natural light and ventilation conditions, following standard commercial cucumber cultivation practices. All other management procedures were conducted in accordance with established greenhouse cucumber production protocols.

The experiment was conducted over four growing seasons, encompassing disease development cycles under different climatic conditions. Environmental data were continuously collected using the M6A-III intelligent greenhouse monitoring and data acquisition system developed by the Facility Horticulture Engineering Center of Shenyang Agricultural University (Equation 8). Average air temperature and relative humidity within the greenhouse were recorded at 5 min intervals.

Disease occurrence was surveyed every three days at 09:00 h using the five-point sampling method, and monitoring continued until the end of the cucumber production cycle. The resulting field disease data were used for model validation.

A completely randomized design (CRD) was adopted for the field experiment (Equation 9). Treatment plots and replicate blocks were randomly distributed within the greenhouse to minimize the potential influence of spatial environmental heterogeneity. Disease assessments were conducted using a randomized five-point sampling procedure.

In the greenhouse experiment, each treatment plot was considered an independent experimental unit with three biological replicates. Environmental heterogeneity within the greenhouse was minimized using randomized complete block design (RCBD), and no fungicide application was performed throughout the study period.

### 2.3 Data collection and processing

#### 2.3.1 Disease data collection

Disease surveys were conducted using the five-point sampling method (Equation 10). Five sampling points were selected in each plot, with 5 cucumber plants surveyed at each sampling point. Disease severity was classified according to the grading standards in the “Cucumber Leaf Disease Severity Classification” (Du et al., 2023), with the severity divided into five levels: Level 0 (no lesions), Level 1 (lesion area ≤ 5% of the leaf area), Level 2 (lesion area 6%-10% of the leaf area), Level 3 (lesion area 11%-25% of the leaf area), and Level 4 (lesion area > 50% of the leaf area). The incidence rate and disease index were calculated using the following formulas:

(1) $$\text{P}=\frac{{\text{N}}_{\text{d}}}{{\text{N}}_{\text{t}}}\times 100\%$$

Where P is the incidence of cucumber diseases (%), N_d_ is the number of diseased plants, and N_t_ is the total number of plants investigated.

(2) $$\text{DI}=\frac{\sum \left({\text{N}}_{\text{i}}\times {\text{S}}_{\text{i}}\right)}{{\text{N}}_{\text{t}}\times {\text{S}}_{\max}}\times 100\%$$

Where DI is the disease index of cucumber diseases; N_i_ is the number of diseased plants at the i-th level; S_i_ is the disease level value corresponding to the i-th level; N_t_ is the total number of plants investigated; S_max_ is the highest level value in the disease grading.

The criteria for determining the first appearance of disease symptoms are as follows: the presence of clearly identifiable typical lesions on the leaf, with the lesion area ≥1 mm^2^. The incubation period is defined as the time interval from the completion of inoculation to the first occurrence of lesions meeting the above criteria, which serves as the target output variable for model prediction. In this study, prediction accuracy is defined as a correct prediction when the error between the predicted incubation period and the actual observed value is within ±1 day.

#### 2.3.2 Environmental data and data preprocessing

A total of 1248 valid data records (N = 1248) were collected in this study, covering both the climate chamber controlled experiment and greenhouse field experiment data. Environmental data include hourly temperature and relative humidity, from which daily average temperature, daily average relative humidity, and the Infection Suitability Index (ISI) were calculated. Disease data include incubation period, incidence rate, and disease index.

Data preprocessing was conducted using the three-sigma rule (3σ) to remove outliers, and linear interpolation was used to fill in the few missing values. All input variables were normalized to the range [0,1] using the Min-Max Scaler to eliminate the influence of units, as shown in the following formula:

(3) $${\text{X}}_{\text{scaled}}=\frac{\text{X}-{\text{X}}_{\min}}{{\text{X}}_{\max}-{\text{X}}_{\min}}$$

Where X represents the original data value; X_min_ represents the minimum value in the data; X_max_ represents the maximum value in the data; X_scaled_ represents the normalized data value.

The dataset was randomly split into training and testing sets at a 7:3 ratio, and 5-fold cross-validation was used to evaluate the model’s stability and generalization ability, reducing the bias caused by a single data split and ensuring the reliability of the results. To avoid input variable redundancy, variance inflation factor (VIF) tests were performed on temperature, humidity, and ISI. The results showed that VIF< 3, indicating no significant multicollinearity. Additionally, an ablation test was conducted, which confirmed that incorporating ISI alongside temperature and humidity significantly improved prediction accuracy, proving that all three variables are valuable for inclusion.

### 2.4 Prediction model construction and evaluation

In this study, average temperature, average relative humidity, and the Infection Suitability Index (ISI) were used as input variables, and the disease incubation period was used as the output variable. Multiple Linear Regression (MLR), Support Vector Regression (SVR), and a Multiple Linear Regression–Support Vector Regression (MLR-SVR) hybrid prediction model were constructed. These models were then compared against Random Forest (RF) and XGBoost to comprehensively validate the model performance and advantages.

#### 2.4.1 Basic model principles

Multiple Linear Regression (MLR) effectively captures the linear trend between environmental factors and disease incubation period, with a simple structure and strong interpretability, making it suitable as a basic fitting framework. Support Vector Regression (SVR) excels at handling complex nonlinear mapping relationships. In this study, a radial basis function (RBF) kernel was selected, and the grid search algorithm was used to optimize the penalty parameter C, kernel function parameter γ, and the insensitive loss parameter ϵ to achieve the optimal fitting performance.

For SVR modeling, the radial basis function (RBF) kernel was applied. Hyperparameters including penalty coefficient C, kernel parameter γ, and epsilon were optimized using grid search combined with 5-fold cross-validation. Final optimal parameters were selected based on minimum validation error. Model performance metrics reported in this study correspond to the average results of 5-fold cross-validation, while independent test set results were used for final verification.

#### 2.4.2 MLR-SVR hybrid model

**Figure 1:**
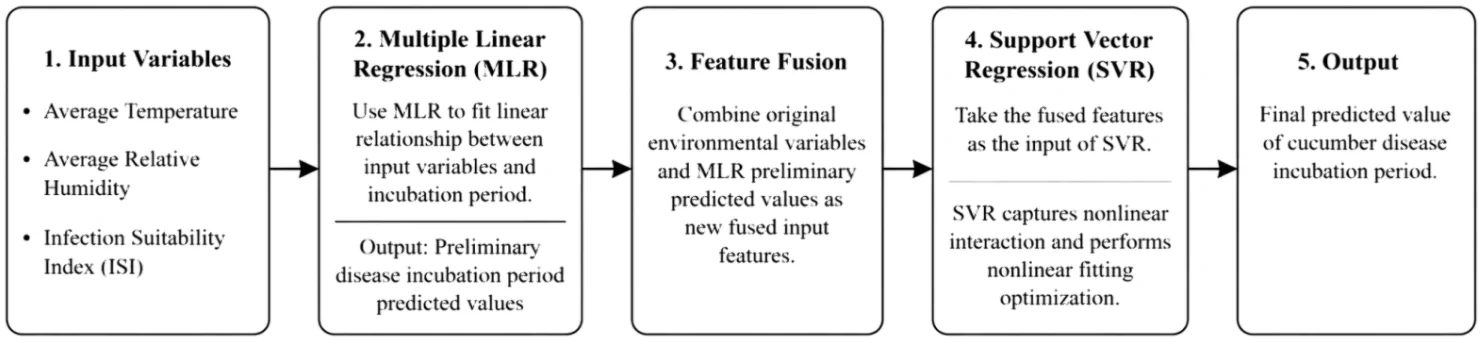
Workflow of the MLR-SVR hybrid model for predicting incubation periods of multiple cucumber diseases. Average temperature, average relative humidity, and the Infection Suitability Index (ISI) were used as input variables. Multiple linear regression (MLR) was first applied to generate preliminary predictions, which were then combined with environmental variables as fused features for support vector regression (SVR) to capture nonlinear relationships and generate the final incubation period prediction.

To account for both linear and nonlinear relationships between greenhouse environmental factors and the disease incubation period, an MLR–SVR hybrid model was established. Initially, multiple linear regression (MLR) was used to model the linear effects of average temperature, average relative humidity, and the Infection Suitability Index (ISI), yielding preliminary predictions of the incubation period. These predictions, together with the original environmental variables, were then used as input features for support vector regression (SVR). The SVR component further captured the nonlinear interactions among environmental factors and disease development. By integrating the strengths of both approaches, the proposed hybrid model combines the interpretability of MLR with the powerful nonlinear learning capability of SVR, resulting in improved predictive performance. The model construction process is shown in **Figure 1**.

#### 2.4.3 Model performance evaluation criteria

In this study, the performance of the prediction models is evaluated using three metrics: Mean Squared Error (MSE), Mean Absolute Error (MAE), and the Coefficient of Determination (R^2^). The models are based on average temperature, average humidity, and the daily values of the integrated Infection Suitability Index as input variables, predicting the duration from cucumber disease infection to the appearance of symptoms.

(4) $$\text{MSE}=\frac{1}{\text{n}}\sum_{\text{i}=1}^{\text{n}}{\left({\text{y}}_{\text{i}}-\hat{{\text{y}}_{\text{i}}}\right)}^{2}$$

(5) $$\text{MAE}=\frac{1}{\text{n}}\sum_{\text{i}=1}^{\text{n}}\left|{\text{y}}_{\text{i}}-\hat{{\text{y}}_{\text{i}}}\right|$$

Where y_i_ is the observed value, $\hat{{\text{y}}_{\text{i}}}$ is the predicted value, and n is the sample size.

(6) $${\text{R}}^{2}=1-\frac{\sum_{\text{î}=1}^{\text{n}}{\left({\text{y}}_{\text{i}}-\hat{{\text{y}}_{\text{i}}}\right)}^{2}}{\sum_{\text{i}=1}^{\text{n}}{\left({\text{y}}_{\text{i}}-\bar{y}\right)}^{2}}$$

Where y_i_ is the observed value, $\hat{{\text{y}}_{\text{i}}}$ is the predicted value, $\bar{y}$ is the mean of the observed values, and n is the sample size.

### 2.5 Infection suitability index model construction

This study introduces the concept of the Infection Suitability Index (ISI) to quantify the comprehensive effect of temperature-humidity coupling on the infection process of cucumber low-temperature, high-humidity diseases. Temperature and humidity are the core environmental factors regulating pathogen infection, and their dynamic coupling characteristics determine the speed of disease onset and the length of the incubation period. A single factor is insufficient to accurately represent the actual infection risk (Zhang and Du, 2025a; Silva et al., 2026).

Existing studies often evaluate the potential for disease infection using environmental suitability functions or risk indices, but these typically rely on empirical thresholds or simple weighting, with limited biological interpretability. This study constructs temperature suitability functions (F_T_) and humidity suitability functions (F_H_) based on the temperature base-point theory and humidity threshold effects. The coupling is calculated in a multiplicative form ( $\text{ISI}={\text{F}}_{\text{T}}\times {\text{F}}_{\text{H}}$) to achieve a quantitative representation of the pathogen’s infection potential in a temperature-humidity coupled environment. Compared to traditional suitability models, this study uses a physiological response function combined with a multiplicative coupling approach to describe the nonlinear synergistic effects of temperature and humidity. This structural approach enhances the biological interpretability and generality of the index, providing core input parameters with clear ecological significance for unified prediction of multiple diseases (Arauz et al., 2010; Neufeld and Ojiambo, 2012).

#### 2.5.1 Temperature suitability sub-model (*F_T_*)

The temperature suitability sub-model is based on the pathogen’s three base-point temperature characteristics, and a Beta function is used to construct a normalized response model (Pivonia and Yang, 2006). By incorporating the biological characteristics of downy mildew (T_min_ = 16 °C, T_opt_ = 20 °C, T_max_ = 24 °C), gray mold T_min_ = 15 °C, T_opt_ = 18 °C, T_max_ = 22 °C), and black spot disease (T_min_ = 17 °C, T_opt_ = 22 °C, T_max_ = 25 °C), the Beta function quantifies the suitability of temperature for pathogen infection. The formula is as follows:

(7) $${\text{F}}_{\text{T}}=\{\begin{array}{cc} 0 & \text{if t}\langle {\text{T}}_{\min}\text{ or t}\rangle {\text{T}}_{\max}\\ {\left(\text{t}-{\text{T}}_{\min}\right)}^{\alpha }{\left({\text{T}}_{\max}-\text{t}\right)}^{\beta } & \text{if }{\text{T}}_{\min}\leq \text{t}\leq {\text{T}}_{\max} \end{array}$$

In the formula: t represents the actual temperature (°C); T_min_, T_opt_, and T_max_ are the minimum, optimal, and maximum temperature thresholds for pathogen infection, respectively; α and β are shape parameters, reflecting the asymmetry of the temperature response curve.

The following values were obtained from experimental data fitting: for downy mildew, α = 1.8 and β = 2.2; for gray mold, α = 2.0 and β = 1.9; for black spot disease, α = 1.7 and β = 2.3. Sensitivity analysis of the parameters showed that the values of these parameters were stable, and small fluctuations did not significantly alter the ISI trend.

The shape parameters (α and β) of the Beta function were estimated using nonlinear least squares fitting based on the controlled environment chamber dataset. The incubation period was used as the response variable, and the optimization objective was to minimize the residual sum of squared errors between observed and modeled infection suitability. Parameter estimation was performed using the Levenberg–Marquardt algorithm implemented in Python (SciPy optimize package).Goodness-of-fit was evaluated using R² and RMSE, confirming the stability of the fitted parameters. In cases where parameters were adopted from previous studies, initial values were set based on published physiological response ranges and further refined using experimental calibration.

#### 2.5.2 Humidity suitability function model (F_H_)

Based on the critical humidity requirement for spore germination of low-temperature, high-humidity diseases (relative humidity ≥ 85%), a piecewise linear function is used to quantify humidity suitability, as shown in the following formula:

(8) $${\text{F}}_{\text{H}}=\{\begin{array}{cc} 0 & \text{if RH}<85\%\\ \left(\frac{\text{RH}-85}{15}\right) & \text{if }85\%\leq \text{RH}\leq 100\% \end{array}$$

In the formula: RH represents the actual relative humidity (%); when RH< 85%, the humidity is insufficient to meet the moisture requirements for spore germination and infection, so F_H_ = 0; when 85% ≤ RH ≤ 100%, FH increases linearly with the humidity up to 1, which aligns with the biological infection characteristics of low-temperature, high-humidity diseases and has been validated in related pathogen studies.

#### 2.5.3 Infection suitability index combined model

The temperature suitability (F_T_) and humidity suitability (F_H_) are integrated in a multiplicative form to construct the ISI comprehensive model, as shown in the following formula:

(9) $$\text{ISI}={\text{F}}_{\text{T}}\times {\text{F}}_{\text{H}}$$

The ISI theoretical value ranges from[0,1], representing the daily inoculation suitability. The higher the ISI, the more suitable the environment is for pathogen infection, and the higher the risk of disease occurrence. The higher values appearing in some figures and tables in the text represent the cumulative ISI, which reflects the cumulative effect of infection under continuously suitable environmental conditions over multiple days. This is calculated using the same formula as the daily ISI, with the only difference being the statistical period.

The cumulative ISI was calculated as the temporal accumulation (running sum) of daily ISI values over consecutive days under continuous infection-conducive conditions.

### 2.6 Parameter sensitivity analysis

To clarify the impact weights of each input parameter on the prediction results of the MLR-SVR hybrid model, a parameter sensitivity analysis was conducted using the control variable method (Wang et al., 2013). The three core input parameters—average temperature, average humidity, and ISI daily average—were selected. Using the experimental measured values of each parameter as the baseline, each parameter was independently varied by ±20% (with a step size of 5%), while keeping the other parameters constant. The sensitivity of the parameters was quantified by calculating the relative change rate of the model prediction values under different parameter change gradients. The formula for calculating the relative change rate is as follows:

(10) $$\text{RCR}=\frac{\left|{\text{y}}_{\text{i}}-{\text{y}}_{0}\right|}{{\text{y}}_{0}}\times 100\%$$

In the formula: RCR represents the relative change rate of the predicted value (%); y_0_ is the model prediction value corresponding to the baseline parameter value; y_i_ is the model prediction value corresponding to the parameter after variation.

### 2.7 Pearson correlation analysis

To clarify the correlation between the Infection Suitability Index (ISI) and disease incubation period, Pearson correlation analysis was performed on the ISI data and corresponding incubation period data for downy mildew, gray mold, and black spot disease. The correlation coefficient (r) was calculated to quantify the linear correlation between the two, and the significance of the correlation was tested using the P-value (with a significance level set to 0.001). Scatter plots and fitting curves were also constructed to visually present the correlation trend. The analysis results were used to verify the biological validity of the ISI index and provide data support for selecting core input parameters for the subsequent multi-disease prediction models.

### 2.8 Statistical analysis

All data statistics and model construction were performed using Python 3.12.3, and charting was done using Origin 2023. Regression model residual analysis and normality tests are presented in the **Supplementary Materials**.

## 3 Results

### 3.1 Performance comparison of different prediction models

To avoid bias introduced by a single dataset split, this study used 5-fold cross-validation to evaluate the prediction performance of all models, with all metrics presented as mean ± standard deviation. Average temperature, average relative humidity, and the daily values of the Infection Suitability Index (ISI) were used as input variables, and the disease incubation period was used as the output variable. Benchmark testing was conducted for Multiple Linear Regression (MLR), Support Vector Regression (SVR), Random Forest (RF), XGBoost, and the MLR-SVR hybrid model. The total sample size was N = 1248, and the training and testing datasets were split in a 7:3 ratio (**Table 1**).

**Table 1:** Prediction performance of different models for the incubation period of downy mildew, gray mold and black spot disease in cucumber.

Disease	Model	R^2^	MSE	MAE
Downy Mildew	MLR	0.915 ± 0.018	0.042 ± 0.005	0.126 ± 0.008
	SVR	0.977 ± 0.009	0.028 ± 0.003	0.098 ± 0.006
	RF	0.958 ± 0.012	0.035 ± 0.004	0.108 ± 0.007
	XGBoost	0.965 ± 0.010	0.031 ± 0.003	0.102 ± 0.006
	MLR-SVR	0.982 ± 0.006	0.018 ± 0.002	0.076 ± 0.005
Gray Mold	MLR	0.896 ± 0.021	0.038 ± 0.005	0.118 ± 0.009
	SVR	0.944 ± 0.013	0.026 ± 0.004	0.105 ± 0.007
	RF	0.923 ± 0.016	0.032 ± 0.005	0.112 ± 0.008
	XGBoost	0.931 ± 0.014	0.029 ± 0.004	0.107 ± 0.007
	MLR-SVR	0.962 ± 0.008	0.021 ± 0.002	0.083 ± 0.004
Black Spot Disease	MLR	0.900 ± 0.019	0.033 ± 0.004	0.109 ± 0.007
	SVR	0.961 ± 0.011	0.024 ± 0.003	0.092 ± 0.005
	RF	0.941 ± 0.014	0.029 ± 0.004	0.101 ± 0.006
	XGBoost	0.949 ± 0.012	0.026 ± 0.003	0.097 ± 0.005
	MLR-SVR	0.975 ± 0.007	0.014 ± 0.002	0.059 ± 0.003

**Figure 2:**
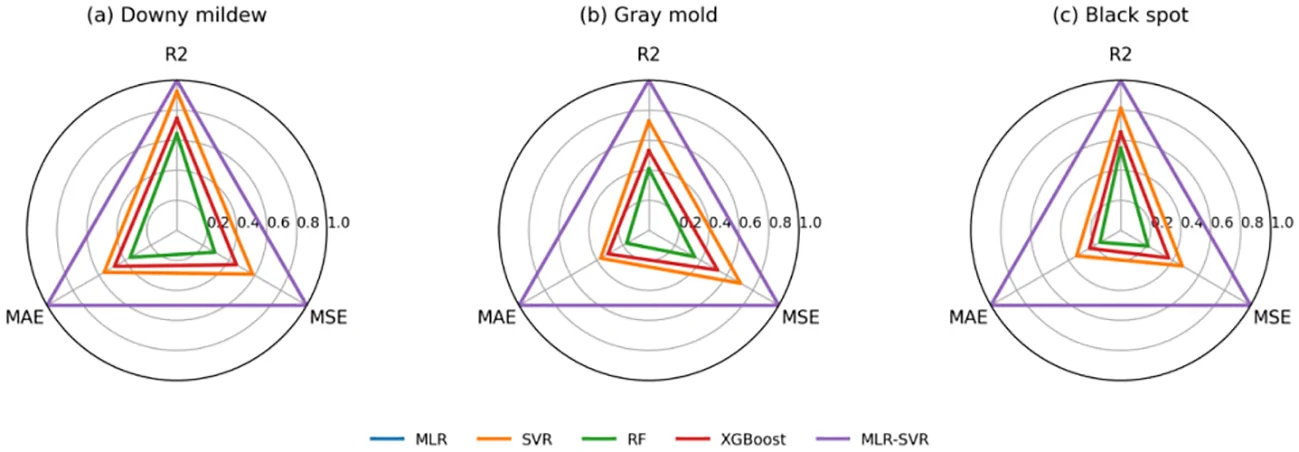
Radar charts comparing the normalized prediction performance of five models for cucumber disease incubation period. Panels show model performance for **(a)** downy mildew, **(b)** gray mold, and **(c)** black spot disease. The compared models include multiple linear regression (MLR), support vector regression (SVR), Random Forest (RF), XGBoost, and the MLR-SVR hybrid model. The evaluation metrics include coefficient of determination (R²), mean squared error (MSE), and mean absolute error (MAE). Higher normalized R² and lower normalized MSE and MAE indicate better prediction performance.

The results of the 5-fold cross-validation show that the MLR-SVR hybrid model performs excellently in predicting all three diseases, with the highest R^2^ values, the lowest MSE and MAE, and significantly better prediction accuracy compared to single models and other machine learning methods (**Figure 2**). Specifically, the R^2^ values of the MLR-SVR hybrid model are as follows: downy mildew 0.982, gray mold 0.962, and black spot disease 0.975. The prediction accuracy (defined as the prediction incubation period with an error within ±1 day of the observed value) ranges from 88.5% to 92.3%, demonstrating that this model can reliably predict the incubation period of three low-temperature, high-humidity diseases in cucumbers. In contrast, the single MLR model has R^2^ values of 0.915, 0.896, and 0.900, and the SVR model has R^2^ values of 0.977, 0.944, and 0.961, indicating that the hybrid model significantly improves prediction performance by integrating linear trends and nonlinear residual fitting.

### 3.2 Correlation between ISI and disease incubation period and biological validity

**Figure 3:**
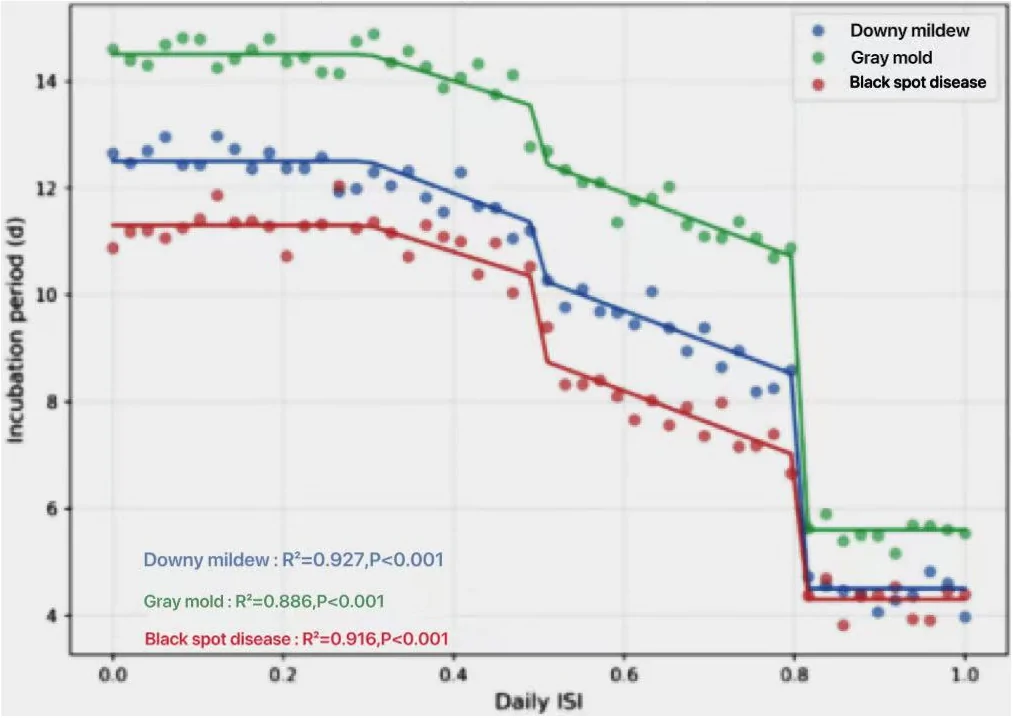
Relationship between daily infection suitability index (ISI) and disease incubation period for three cucumber diseases. Scatter points represent observed incubation periods under different temperature–humidity treatments, and fitted curves show the response trends for downy mildew, gray mold, and black spot disease. Daily ISI ranges from 0 to 1, with higher values indicating more favorable environmental conditions for pathogen infection. A shorter incubation period at higher ISI values indicates stronger infection suitability under low-temperature and high-humidity conditions.

To validate the biological significance of the ISI, this study calculated the Pearson correlation coefficients (r) between daily ISI values (ranging from [0,1]) and the incubation periods of the three diseases, followed by significance testing (P< 0.001). The results showed that daily ISI was highly negatively correlated with the incubation periods of downy mildew, gray mold, and black spot disease (r = -0.963, -0.941, -0.957; R^2^ = 0.927, 0.886, 0.916; N = 1248), indicating that the ISI index accurately reflects the changes in disease incubation periods (**Figure 3**).

Additionally, correlation analysis between ISI and spore germination rate and lesion expansion rate in the climate chamber experiment showed significant positive correlations (P< 0.001), further proving that higher ISI values correspond to stronger pathogen activity, indicating clear biological significance. Through segmental analysis, it was found that the response of ISI to incubation period follows a typical segmented pattern: ISI = 0.3–0.5 (low suitability): the incubation period is longer and decreases slowly; ISI = 0.5–0.8 (moderate to high suitability): the incubation period shortens rapidly; ISI > 0.8 (high suitability): the incubation period tends to stabilize. The incubation periods for downy mildew were approximately 4.2–5.3 days, for gray mold 5.1–6.2 days, and for black spot disease 3.8–4.7 days.

### 3.3 Effect of temperature-humidity combinations on disease inoculation suitability

**Figure 4:**
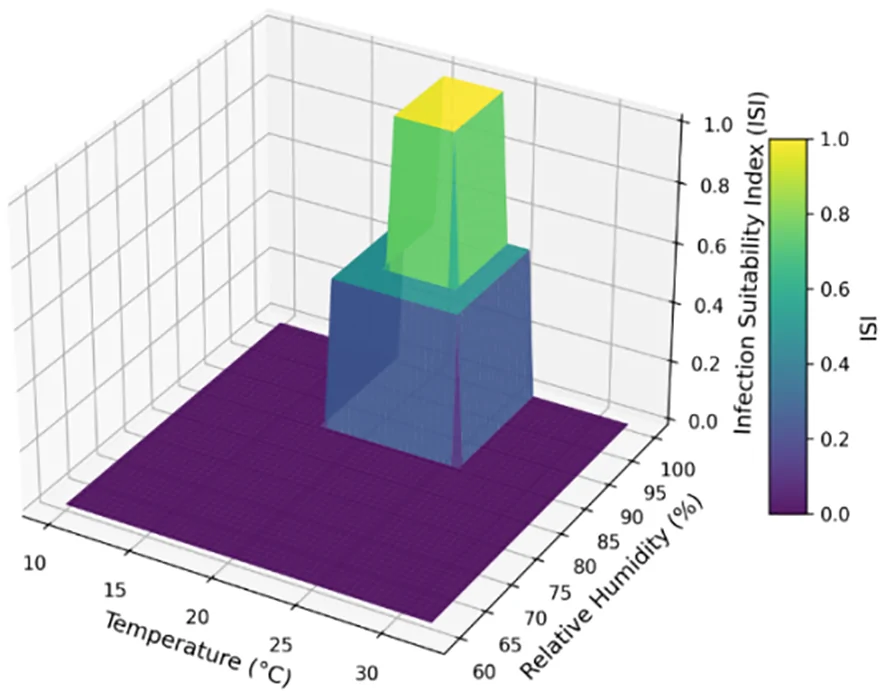
Three-dimensional response surface showing the coupling effect of temperature and relative humidity on the Infection Suitability Index (ISI). The x-axis represents temperature, the y-axis represents relative humidity, and the z-axis or color gradient represents ISI values. Higher ISI values indicate environmental conditions more favorable for pathogen infection. The surface illustrates that disease infection suitability increases only when both temperature and relative humidity fall within suitable ranges, supporting the nonlinear coupling effect of temperature and humidity.

Through three-dimensional surface analysis of temperature-humidity coupling, the impact of different temperature-humidity conditions on the ISI and disease inoculation potential was observed (**Figure 4**). The results showed that: at temperatures of 20–24 °C and relative humidity >90%, ISI reached its maximum value, indicating the highest disease risk; at temperatures of 18–26 °C and humidity >85%, ISI was in the moderate range, corresponding to moderate disease risk; at temperatures< 14 °C or > 30 °C and humidity< 75%, ISI approached 0, with nearly no disease occurrence. The response trends for the three diseases were consistent, but the optimal ranges exhibited pathogen specificity, which aligns with their biological characteristics.

### 3.4 Sensitivity analysis of model input parameters

**Figure 5:**
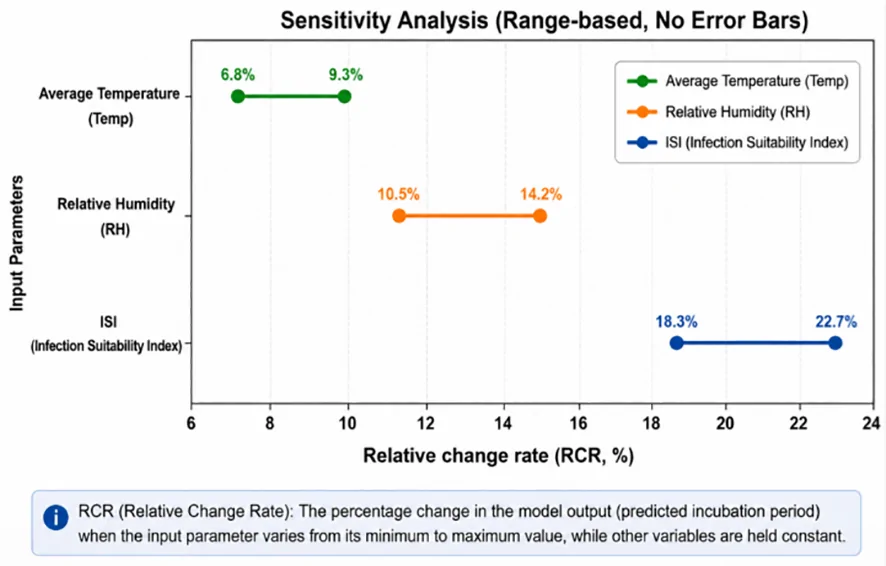
Sensitivity analysis of the three input parameters in the MLR-SVR hybrid model. Average temperature, average relative humidity, and daily Infection Suitability Index (ISI) were independently varied within the tested range while the other variables were held constant. The relative change rate (RCR, %) represents the percentage change in the predicted incubation period caused by variation in each input parameter. Larger RCR values indicate greater sensitivity of model predictions to the corresponding parameter.

The control variable method analysis based on the original actual units (°C, %) showed that the sensitivity of ISI daily average was the highest: with a ±20% fluctuation in the parameters, the prediction values changed by ±18.3% to ±22.7%; average relative humidity showed the next highest sensitivity, with a change of ±10.5% to ±14.2%; average temperature had the lowest sensitivity, with a change of ±6.8% to ±9.3% (**Figure 5**).

The results indicate that ISI is the core variable affecting prediction accuracy, and in practical applications, the accuracy of ISI and humidity data should be prioritized.

## 4 Discussion

### 4.1 Temperature-humidity coupling quantification method analysis

An Infection Suitability Index (ISI) was constructed based on the cardinal temperature and humidity thresholds governing pathogen infection and development. Specifically, temperature suitability was quantified using a Beta response function, whereas humidity suitability was represented by a piecewise linear function. The combined effects of temperature and humidity were then integrated through a multiplicative framework to capture their nonlinear interaction. Compared with traditional disease risk indicators, such as the Thom Index and Hutton Index, the proposed ISI is grounded in pathogen biological mechanisms and provides greater quantitative precision in describing environmental suitability for infection. Consequently, it can serve as a robust indicator for the quantitative assessment of disease risk under the low-temperature and high-humidity conditions commonly encountered in greenhouse production systems (Neufeld et al., 2017; Sun et al., 2017a; Zhong et al., 2020; Ren et al., 2025).

In contrast to studies like González-Domínguez et al., 2023; Castaño-Serna et al., 2025; Dancey, 2018 which focus on single diseases and rely on empirically set thresholds to construct predictive models, this study’s ISI is based on the physiological processes of pathogens, rather than purely empirical fitting. It allows for unified representation of downy mildew, gray mold, and black spot disease through parameter replacement, overcoming the limitation of traditional models that are only applicable to single diseases. Additionally, by distinguishing between daily values and cumulative values, this approach improves the applicability and interpretability of the index across different monitoring scenarios.

Furthermore, ISI clearly differentiates between daily values and cumulative values. The daily values range from [0,1], while the cumulative values reflect the cumulative effect under continuously suitable conditions, providing interpretability and practicality in various scenarios. Pearson correlation analysis shows that daily ISI is highly negatively correlated with the incubation periods of all three diseases (P< 0.001), and positively correlated with spore germination rate and lesion expansion rate, further validating the biological effectiveness of ISI. It also shows that the temperature range of 18–26 °C and relative humidity >85% is a common high-risk range for all three diseases, which is consistent with existing plant epidemiological conclusions, indicating that ISI can scientifically represent the environmental driving mechanisms of multiple disease outbreaks in greenhouses (Miller et al., 2003; Clarkson et al., 2014; Sun et al., 2017a; Zhao et al., 2020).

### 4.2 Predictive advantages and rationality of the MLR-SVR hybrid model

The MLR-SVR hybrid model first captures the linear trend through MLR and generates preliminary predictions. These predictions are then fused with the original environmental variables and used as input features for SVR, enabling the model to further learn nonlinear interactions between environmental conditions and disease incubation period. In the prediction of incubation periods for the three diseases, this model demonstrated excellent performance, with R^2^ values ranging from 0.962 to 0.982, significantly outperforming single MLR, SVR, and traditional machine learning models such as Random Forest and XGBoost.

MLR provides clear linear contribution explanations, ensuring model interpretability; SVR captures the nonlinear features of greenhouse environments and disease responses, compensating for the limitations of single models. It is important to note that MLR is not redundant, but is used to separate linear trends and reduce the complexity of nonlinear fitting, thereby enhancing the robustness and stability of the hybrid model. Logistic regression was not adopted because this study focuses on continuous numerical prediction tasks, where the objective is to predict incubation period length rather than classification (Alwee et al., 2013; Keshtegar et al., 2021; Sabouri et al., 2024).

Collinearity tests and ablation experiments further demonstrated that the inclusion of temperature, humidity, and ISI significantly improved prediction accuracy, with no significant redundancy between variables, indicating that the model structure is scientifically sound and reasonable.

### 4.3 Limitations and future work

Although the proposed ISI framework and MLR-SVR hybrid model showed good predictive performance, several limitations should be acknowledged. First, the experimental data were obtained from a single region (Shenyang Agricultural University Research Base) and a single greenhouse type, and the applicability of the model to different climatic zones and cultivation systems requires further validation. This limitation is consistent with the insufficient regional adaptability of greenhouse environmental models reported by Guo et al. (2021). In addition, detached-leaf assays may not fully represent whole-plant responses under practical greenhouse conditions, and external validation across multiple regions and greenhouse systems has not yet been conducted.

Second, the current model considers only temperature and relative humidity, while other important factors, such as cultivar resistance, pathogen inoculum intensity, leaf wetness duration, and canopy structure, were not included. Future studies could incorporate these variables into the ISI framework using the multi-source information fusion strategy proposed by Cao et al. (2025), while expanding multi-location experiments and integrating IoT-based environmental monitoring to improve model robustness and applicability. Furthermore, because the prediction performance for gray mold (R² = 0.962) was slightly lower than that for the other two diseases, the nonlinear humidity response function proposed by Silva et al. (2026) could be incorporated to further optimize the humidity suitability sub-model (FH) and improve prediction accuracy.

## 5 Conclusion

This study proposes a temperature-humidity coupled Infection Suitability Index (ISI) and an MLR-SVR hybrid model for predicting the incubation periods of downy mildew, gray mold, and black spot disease in greenhouse cucumbers. ISI quantifies temperature suitability using a Beta function, quantifies humidity suitability with a piecewise linear function, and achieves nonlinear coupling of temperature and humidity in a multiplicative form, creating a universal calculation framework adaptable to multiple diseases while distinguishing between daily values and cumulative values.

In terms of model prediction, the MLR-SVR hybrid model demonstrated excellent performance in predicting the incubation periods of all three diseases (R^2^ = 0.962–0.982, prediction accuracy = 88.5%–92.3%), significantly outperforming single MLR, SVR, and classic machine learning methods such as Random Forest and XGBoost.

This study also identifies high-risk temperature-humidity ranges for multiple diseases in greenhouses (temperature 18–26 °C, relative humidity > 85%), providing a quantitative basis for early disease warning and precise control. However, this study has some limitations: the model only uses temperature, humidity, and ISI as inputs and does not consider other potential factors influencing disease development, such as leaf wetness duration, light, and canopy density; all data were sourced from a single solar greenhouse, and the model’s generalizability in other geographic areas, different cultivars, or cultivation practices still needs to be verified; the prediction accuracy for gray mold is slightly lower than for the other diseases, suggesting that its environmental response is more complex.

## Data Availability

All datasets generated and analyzed in this study are included within the article and its supplementary materials. Readers who require additional raw data can contact the corresponding author upon reasonable request.

## Supplementary material

Supplementary Table 1 Temperature and humidity treatments in the artificial climate chamber simulation experiment for disease inoculation.
